# Commentary: Attitudes of Local Population of Tourism Development Impacts

**DOI:** 10.3389/fpsyg.2021.727287

**Published:** 2021-08-25

**Authors:** Álvaro Dias, Mafalda Patuleia

**Affiliations:** ^1^Universidade Lusófona, ECEO/TRIE, Lisbon, Portugal; ^2^ISCTE-IUL, DMOGG, Lisbon, Portugal; ^3^GOVCOPP, Aveiro, Portugal

**Keywords:** destination development, tourism entrepreneurship, COVID-19, competitiveness, place attachment

## Introduction

The impact of tourism on small communities located in peripheral areas, many of them rural, was the subject of the study conducted by Linderová et al. (2021). The authors advocate the critical participation of the local community in tourism development. This is a very important issue that has been widely debated in the context of the pandemic and post-pandemic resulting from COVID-19. Indeed, this strongly adverse context for tourism is also a time to rethink pre-pandemic tourism, massive, and generally damaging to the tourism destination, toward more sustainable and inclusive approaches for the local community (Hall et al., 2020).

Thus, this commentary aimed to complement the perspective of the authors on the benefit of local communities from tourism development. More specifically, it aimed to align the article with the core of this special issue, highlighting the role that lifestyle entrepreneurs have in distributing the value generated by tourism among local actors.

## A Look at the Demand Side

Despite the recognition that mass tourism is not sustainable, most destinations do not present a different approach to deal with the crisis generated by the pandemic (Fisher and Wilder-Smith, 2020). Pursuing a more sustainable path points to the definition of long-term strategies and not only immediate solutions such as communicating that the destination is healthy (Gössling et al., 2020). This means seeking solutions that integrate the perspectives of all stakeholders in the destination and that creativity (Linderová et al., 2021) and ensure a better distribution of value (Gössling et al., 2020). As stated by Linderová et al. (2021), the local community should perceive the benefits of tourism in their daily lives. This idea advocated by the authors points to the effect on local entrepreneurship, as they recognize that accommodation, catering, retail, and cultural activities could benefit from increased tourism. Furthermore, this local business associated with the place identity is the basis for their competitiveness and differentiation, since they are selling unique experiences based on local traditions, narratives, or other idiosyncratic cultural experiences (Dias et al., 2020b). As stated by Linderová et al. (2021), tourism development is also seen as a basis for cultural heritage revitalization.

The involvement of the local community also considers looking at the supply side so that the community can benefit from tourism and so that there is a better distribution of value as advocated by Hall et al. (2020). There are two main reasons: First, the stimulation of entrepreneurial activity through tourism growth contributes to increase the confidence of rural communities in new business creation, facilitate access to distribution channels, and develop a shared vision regarding the development path to follow (Dias et al., 2020a). Second, destination differentiation relies on the innovation locally generated, which depends on the ability to attract creative businesses (De Bruin and Jelinčić, 2016), in particular, entrepreneurs who are motivated by this innovative spirit (Carlsen et al., 2008), as argued by Linderová et al. Tourism lifestyle entrepreneurs (TLEs) fit perfectly into this profile as they are associated with creative businesses with a strong place and community attachment (Thomas et al., 2011; Yachin, 2019).

## Lifestyle Entrepreneurship

Sustainable tourism development requires special attention to the preservation of social and environmental dimensions in the destination. The local identity and lifestyle are aspects that TLEs are particularly interested in preserving (Bosworth and Farrell, 2011), as this is the basis of their choice of business (and lifestyle) location, associated with the quality of life they seek, as stated by Linderová et al. (2021). The position defended by these authors is that also the “new tourists,” prefer holidays oriented to their interest and buy tourism products that are more based on nature, authenticity, and experience. This is precisely the type of tourism products associated with TLEs, as Shaw and Williams (2009); Bosworth and Farrell (2011), and Dias et al. (2020b) point out.

In the context of the study developed by Linderová et al., fostering entrepreneurship in rural destinations such as Predhradí suggests retaining native investors and attracting entrepreneurs from other regions and abroad who are attracted by the local lifestyle and the natural, landscape, and social context, which corresponds to the framework proposed for TLEs by (Wang et al., 2019).

## Discussion and Conclusions

The commentary aimed to complement the study by Linderová et al. by detailing the role that the supply side plays in aligning destination community involvement with tourism development in rural regions. At the same time, it was intended to bridge the gap with the core of this special issue by understanding the link between the study and TLEs. It is possible to perceive that the involvement of communities and the distribution of the values generated by tourism require the promotion of local entrepreneurship. In addition, the study developed by Linderová et al. (2021) highlights the need to promote creativity and innovation in the rural destination, an area in which TLEs may play a relevant role, for two reasons. The first, by their expressiveness, given that in a recent study (LEO, 2021) it was found that about 43% of small business owners in tourism reveal pursuing lifestyle objectives. Second, due to the strong association of these entrepreneurs with creativity and innovation, they are likely to increase destination competitiveness. However, it is also important to recognize that this path is not an easy one due to the limitations of these entrepreneurs, which must be addressed. Indeed, due to the low barriers to entry in some tourism businesses, these entrepreneurs show low qualifications (Ioannides and Petersen, 2003), limited resources, and low management skills (Cooper, 2015).

## Author Contributions

AD and MP are responsible in equal parts for the writing of the text. All authors contributed to the article and approved the submitted version.

## Conflict of Interest

The authors declare that the research was conducted in the absence of any commercial or financial relationships that could be construed as a potential conflict of interest.

## Publisher's Note

All claims expressed in this article are solely those of the authors and do not necessarily represent those of their affiliated organizations, or those of the publisher, the editors and the reviewers. Any product that may be evaluated in this article, or claim that may be made by its manufacturer, is not guaranteed or endorsed by the publisher.
